# Allosteric modulation of adenosine A1 and cannabinoid 1 receptor signaling by G‐peptides

**DOI:** 10.1002/prp2.673

**Published:** 2020-10-30

**Authors:** Anja M. Touma, Rabia U. Malik, Tejas Gupte, Sivaraj Sivaramakrishnan

**Affiliations:** ^1^ Department of Genetics, Cell Biology, and Development University of Minnesota Minneapolis MN USA

**Keywords:** adenosine‐5’‐(N‐ethylcarboxamide), GTP‐binding proteins, N(6)‐cyclopentyladenosine, receptor, adenosine A1, receptor, cannabinoid, CB1, SCH 442 416, signal transduction

## Abstract

While allosteric modulation of GPCR signaling has gained prominence to address the need for receptor specificity, efforts have mainly focused on allosteric sites adjacent to the orthosteric ligand‐binding pocket and lipophilic molecules that target transmembrane helices. In this study, we examined the allosteric influence of native peptides derived from the C‐terminus of the Gα subunit (G‐peptides) on signaling from two Gi‐coupled receptors, adenosine A1 receptor (A_1_R) and cannabinoid receptor 1 (CB_1_). We expressed A_1_R and CB_1_ fusions with G‐peptides derived from Gαs, Gαi, and Gαq in HEK 293 cells using systematic protein affinity strength modulation (SPASM) and monitored the impact on downstream signaling in the cell compared to a construct lacking G‐peptides. We used agonists N^6^‐Cyclopentyladenosine (CPA) and 5’‐*N*‐Ethylcarboxamidoadenosine (NECA) for A_1_R and 2‐Arachidonoylglycerol (2‐AG) and WIN 55,212‐2 mesylate (WN) for CB_1_. G‐peptides derived from Gαi and Gαq enhance agonist‐dependent cAMP inhibition, demonstrating their effect as positive allosteric modulators of Gi‐coupled signaling. In contrast, both G‐peptides suppress agonist‐dependent IP_1_ levels suggesting that they differentially function as negative allosteric modulators of Gq‐coupled signaling. Taken together with our previous studies on Gs‐coupled receptors, this study provides an extended model for the allosteric effects of G‐peptides on GPCR signaling, and highlights their potential as probe molecules to enhance receptor specificity.

## Graphical abstract

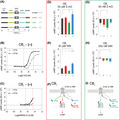

**Abbreviations**
2‐AG: 2‐Arachidonoylglycerol
A_1_R: Adenosine type 1 receptor
A_2A_R: Adenosine type 2A receptor
A_2B_R: Adenosine type 2B receptor, β2‐AR, β2‐adrenergic receptor
CB_1_: cannabinoid type 1 receptor
CPA: N^6^‐Cyclopentyladenosine
D_1_R: Dopamine receptor
G protein: GTP‐binding protein
GPCR: G protein‐coupled receptor
IP: Inositol Phosphate
NECA: 5’‐N‐Ethylcarboxamidoadenosine
PTX: pertussis toxin
SPASM: systematic protein affinity strength modulation
V_1A_‐R: Vasopressin 1A receptor
WN: WIN 55,212‐2 mesylate

## 1 INTRODUCTION

G protein‐coupled receptors (GPCRs) have been the most successful class of drug targets in clinical medicine, due in part to their widespread distribution and important roles in physiology.^1^ The pharmacological success of GPCRs derives from their selective coupling to specific heterotrimeric G proteins, triggering the corresponding physiological response. Recent drug discovery efforts have focused on the development of allosteric modulators for GPCRs.^2^ Allosteric modulators have the potential to increase receptor specificity by targeting sequence motifs unique to receptor family subtypes and isoforms. Furthermore, allosteric modulators require the presence of an orthosteric ligand, providing physiological context‐dependent control of GPCR signaling.^3^ Therefore, compared to orthosteric ligands, large doses of allosteric modulators can be administered with a lower risk of target‐based toxicity.^2^ An emerging target site for allosteric modulators is the GPCR‐G protein‐binding interface. The GPCR‐G protein‐binding interface contains sequence divergent structural elements including three intracellular loops and the GPCR C‐tail.^4^ However, the intrinsically disordered nature of the loop and C‐tail, combined with the potential for binders in these regions to disrupt GPCR‐G protein coupling has limited efforts to rationally design allosteric modulators that target the GPCR‐G protein interface.^5^

In this study, we examine the potential for the G protein α subunit C‐terminus (G‐peptide) to serve as an allosteric modulator of GPCR signaling. The G‐peptide is a well‐established determinant of GPCR‐G protein coupling selectivity.^6^, ^7^ The G‐peptide interacts at the cytosolic GPCR‐G protein interface, which is distinct from the orthosteric ligand‐binding pocket. The GPCR interaction with a cognate G‐peptide triggers nucleotide exchange in the Gα subunit (GDP to GTP) resulting in G protein activation and downstream signaling. While interactions with noncognate G‐peptides do not precipitate G protein activation, we have recently shown that noncognate interactions alter receptor conformation resulting in enhanced ligand efficacy.^8^, ^9^ Previous studies show that while the noncognate G‐peptide interactions are transient, the GPCR conformational state persists following dissociation resulting in the allokairic modulation (AKM) of downstream signaling.^8^, ^9^ Allokairic modulators bind asynchronously with the ligand and rely on the temporal persistence of GPCR conformation to exert their influence on orthosteric ligand efficacy.^9^ Our previous studies focused on the Gs‐coupled β2‐adrenergic (β2‐AR) and dopamine (D1R) receptors, which show enhanced cyclic AMP generation in the presence of a noncognate Gq protein. Likewise, the Gq‐coupled V1 vasopressin receptor (V1R) shows enhanced IP_1_ levels in the presence of the noncognate Gs protein.^8^ In this study, we examine the potential for allokairic modulation of two canonical Gi‐coupled receptors, adenosine type 1 (A1R) and cannabinoid type 1 (CB1) using G‐peptides derived from Gs, Gi, and Gq subtypes.

While β2‐AR and D_1_R principally signal through Gs, and A_1_R and CB_1_ primarily signal through Gi. However, A_1_R and CB_1_ display signaling through multiple G proteins with A_1_R signaling through Gi and Gq, and CB_1_ signaling through Gi, Gq, and Gs.^10^, ^11^, ^12^, ^13^ CB_1_, the most widely expressed GPCR in the central nervous system, primarily signals through Gi producing euphoria and analgesia upon binding tetrahydrocannabinol (THC) in the brain.^14^, ^15^ CB_1_ has also been shown to signal through Gq in human embryonic kidney (HEK) 293 cells after treatment with WIN55,212‐2 (WN) ^12^ and through Gs in rat globus pallidus, HEK 293, COS‐7, CHO, and 3T3 cells after treatment with WN.^10^, ^13^, ^16^ However, the physiological effects of CB_1_ signaling through Gs, Gq, and non‐G protein‐mediated pathways is less clear since there have not been biased ligands identified that specifically target these pathways. A_1_R is another example of a promiscuous receptor that can activate different signal transduction pathways in an agonist‐dependent manner. A_1_R is ubiquitously expressed and most well known for being antagonized by caffeine, producing stimulant effects.^17^ While A_1_R canonically signals through Gi, there is evidence that A_1_R has a diverse G protein‐activating profile where A_1_R can adopt agonist‐specific conformations, arising from small changes in ligand structure, which lead to the differential activation of G proteins including Gi and Gq.^11^ This promiscuity of coupling in these canonical Gi receptors allows us to examine the allosteric effects of the G‐peptide on multiple G protein signaling pathways.

The goal of this focused study is to examine the allosteric effects of G‐peptides derived from three distinct Gα C‐termini peptides (Gαs, Gαi, and Gαq) on signaling from two promiscuous Gi‐coupled receptors (A_1_R and CB_1_). The C‐termini of three G proteins, Gαs, Gαi, and Gαq, will be referred to as s‐pep, i‐pep, and q‐pep (or collectively as G‐peptides) throughout this manuscript. We expressed A_1_R and CB_1_ fusions with the s‐, i‐, or q‐pep in HEK 293 cells using systematic protein affinity strength modulation (SPASM) and monitored the impact on downstream signaling in the cell compared to a construct lacking this G‐peptide, referred to henceforth as no‐pep. We have extensively reported on this SPASM technique, which allows systematic control of the intramolecular interaction between a GPCR and a G‐peptide.^6^, ^8^, ^18^, ^19^ This technology allows us to directly compare the influence of different G‐peptides on the cognate G protein signaling pathways in cells. While this is a tethered system, we have shown that these engineered GPCR constructs yield similar results to reconstituted systems of GPCR membranes and recombinant G proteins with regards to allokairic modulation of G protein activation.^8^, ^19^ Hence, despite the synthetic nature of our approach, it provides insight into the impact of receptor interactions with G‐peptides on downstream signaling.

To investigate the allosteric effects of G‐peptides on Gi‐coupled receptors, we used N ^6^ ‐Cyclopentyladenosine (CPA) and 5’‐ *N* ‐Ethylcarboxamidoadenosine (NECA) for A_1_R and 2‐Arachidonoylglycerol (2‐AG) and WIN 55,212‐2 mesylate (WN) for CB_1_. Our current study confirms what we previously found in Gs‐coupled receptors β_2_‐AR and D_1_‐R, where s‐pep and q‐pep positively modulate canonical Gs signaling.^8^ cAMP response at high concentrations of 2‐AG and WN is enhanced by q‐pep (~30% and 95% increase in cAMP, respectively). Likewise, cAMP stimulation by WN at CB_1_ is enhanced by s‐pep (~40% increase). In contrast, i‐pep diminishes cAMP response from CB_1_ for both 2‐AG and WN (30 and 50% decreases, respectively). At low concentrations of 2‐AG, WN, and CPA (nmol/L) we observed inhibition of cAMP, associated with signaling through Gi. We found that the presence of q‐pep or i‐pep enhanced canonical Gi signaling in A_1_R after activation by CPA (~35% increase), and in CB_1_ after activation by WN (~700% increase) and 2‐AG (~125% increase), respectively. These findings extend our previously reported allosteric effects of G‐peptides to Gi‐coupled signaling.^8^, ^9^ At high concentrations of 2‐AG, WN, CPA, or NECA (μmol/L), stimulation of inositol phosphate (IP_1_) is observed, associated with signaling through Gq. We found that the presence of different G‐peptides universally inhibits IP_1_ signaling through Gq (decreases ranging from 30% to 65%), with the exception of s‐pep (~50% increase) on CB_1_ following activation by WN. Taken together, our data provide an extended model for the allosteric effects of distinct G‐peptides on signaling through Gs, Gi, and Gq pathways and highlight the ability of G‐peptides to differentially impact signaling in a receptor and ligand‐dependent manner.

## 2 MATERIALS AND METHODS

### 2.1 Reagents and buffers

5’‐*N*‐Ethylcarboxamidoadenosine (NECA), pertussis toxin (PTX), and forskolin were purchased from Sigma‐Aldrich. 2‐Arachidonoylglycerol (2‐AG), N^6^‐Cyclopentyladenosine (CPA), WIN 55,212‐2 mesylate (WN), SCH 442416 (SCH), and PSB 1115 (PSB) were purchased from Tocris. cDNA encoding Gαi_2_ isoform 1, Gαq, and the long splice variant of Gαs were acquired from GE (Open Biosystems). Human A_1_R was acquired from DNASU Plasmid Repository. *Mus musculus* CB_1_ was acquired from transOMIC technologies. DNA transfection reagents X‐tremeGENE HP and Mirus‐LT DNA were purchased from Roche and Mirus, respectively. Buffer A is phosphate‐buffered saline (PBS pH 7.4; Gibco^TM^), 800 μmol/L ascorbic acid, and 0.2% dextrose (w/v). Buffer B (Stimulation Buffer 2; Cisbio) is 10 mmol/L HEPES, 1 mmol/L CaCl_2_, 0.5 mmol/L MgCl_2_, 4.2 mmol/L KCl, 146 mmol/L NaCl, 5.5 mmol/L glucose, 50 mmol/L LiCl_2_, pH 7.4.

### 2.2 Molecular cloning

For mammalian HEK 293 expression, all GPCR and Gα constructs were cloned into a PCDNA5/FRT vector (ThermoFisher). GPCR sensors were cloned with a modular scheme. Each GPCR sensor contained (from N‐ to C‐terminus): a full length GPCR (A_1_R or CB_1_), mCitrine, 10 nm ER/K linker, mCerulean, and a Gα subunit C‐terminal peptide corresponding to Gαs, Gαi, Gαq, (s‐pep, i‐pep, or q‐pep, respectively) or a control peptide (no‐pep), consisting of repeating (Gly‐Ser‐Gly)_4_ residues. A (Gly‐Ser‐Gly)_4_ linker was inserted between all protein domains as part of the primer sequence to allow for free rotation between domains. All sensors also contained either an N‐terminal HA‐tag or a His‐tag. All constructs were confirmed by sequencing.

### 2.3 Mammalian cell preparation and sensor expression

HEK293T‐Flp‐In (HEK293T, ThermoFisher) cells were cultured in DMEM media (ThermoFisher) supplemented with 10% FBS (v/v) (Millipore Sigma), 4.5/gL D‐glucose, 1% Glutamax (ThermoFisher), 20 mmol/L HEPES, pH 7.5 at 37°C in a humidified atmosphere at 5% CO_2_. HEK293T cells were plated onto six‐well tissue culture treated plates at ~30% confluence. Cells were transfected 16‐20 hours later with X‐tremeGENE HP DNA transfection reagent. Transfection conditions including the amount of DNA (1.4‐4 μg DNA + 4.2‐6 μL reagent) and the length of transfection (control no‐pep sensors: 18‐24 hours; sensors containing s‐, i‐, or q‐pep: 22‐32 hours) were optimized to consistently yield equivalent levels of sensor expression across different conditions. Where indicated, 12 hours after transfection, cells were incubated with 100 ng/mL PTX for 16 hours. Experiments were conducted at 60%‐80% transfection efficiency (evaluated on a Nikon tissue‐culture microscope enabled with fluorescence detection using 20x and 40× magnification). At the time of the experiment, 60%‐90% of transfected cells expressed predominantly plasma membrane localized sensor with minimal localization to the intracellular compartments. Sensor integrity, localization, and sensor expression were tracked for all experiments to ensure consistency. Each experiment was performed at equivalent sensor expression and matched OD of the cell suspension using the following steps. Cells were first resuspended by gentle pipetting into their original media, spun down (350 g, 3 minutes), and washed once with Buffer A or B for cAMP or IP_1_ assays, respectively. Subsequently, cells were resuspended in an appropriate volume of the same buffer to reach a 0.3 OD measured at A_600 nm_. Sensor expression was measured by mCitrine fluorescence. mCitrine fluorescence was held within 1.6‐2.4 × 10^6^ counts‐per‐second (cps) for a cell OD of 0.3. Sensor integrity was confirmed by measuring the mCitrine (Horiba Fluoromax‐4; excitation 490 bandpass 8 nm; emission range 500‐600 bandpass 4 nm; emission maximum 525 nm) to mCerulean fluorescence ratio (excitation 430 bandpass 8 nm; emission range 450‐600 bandpass 4 nm; emission maximum 475 nm). Experiments were conducted at mCitrine to mCerulean fluorescence ratio of 1.7‐2.1.

### 2.4 cAMP assays

HEK293T cells expressing indicated sensor were harvested 28‐32 h posttransfection (X‐tremeGENE HP) to assess cAMP levels using the bioluminescent cAMP Glo assay (Promega). Cells were gently suspended in their original media, counted using a hemocytometer, and spun down (350 g, 3 minutes). Cells were resuspended in an appropriate volume of Buffer A to reach 4 × 10^6^ cells/mL density. Cell suspensions were aliquoted into 384‐well opaque plates (5 μL per well). Where indicated, cells were preincubated with 100 nmol/L of the adenosine type 2A receptor (A2AR) selective antagonist, SCH 442416 (SCH), and 1 μmol/L of the adenosine type 2B receptor (A2BR) selective antagonist, PSB 1115 (PSB) in 10 μmol/L forskolin for 15 minutes at 37°C. Cells were incubated with CPA or NECA (for A_1_R) or 2‐AG or WN (for CB_1_) for 15 minutes with 10 μmol/L forskolin at 37°C. Subsequently, cells were lysed and the protocol was followed according to the manufacturer's recommendation (Promega). Luminescence was measured using a microplate reader (SpectraMax M5e, Molecular Devices). cAMP levels were evaluated by subtracting relative luminescence units (RLUs) in the absence and presence of agonists. Each experiment was performed in quadruplicate and independently repeated at least three times (N > 3). For experiments involving comparisons between multiple sensors, equivalent sensor expression was first verified using fluorescence measurements (see previous section) and data for all four sensors were collected together (Figure S3).

### 2.5 IP_1_ assays

HEK293T cells expressing the indicated sensor were harvested 28‐32 h posttransfection (X‐tremeGENE HP) to assess IP_1_ levels using the IP‐One HTRF assay kit (Cisbio). Cells were gently suspended in their original media, counted using a hemocytometer, and spun down (350 g, 3 minutes). An appropriate volume of Buffer B (StimB buffer) was added to reach 3 × 10^6^ cells/mL density. Where indicated, cells were preincubated with 1 μmol/L of the A_2B_R selective antagonist, PSB 1115 (PSB) for 30 minutes at 37°C. Cells were incubated with 100 μmol/L of CPA or NECA (for A_1_R) or 100 μmol/L of 2‐AG or WN (for CB_1_) at 37°C for a total incubation time of 30 or 120 minutes. The manufacturer's protocol was modified to achieve a high signal to noise ratio as follows: 70 μL of suspension was incubated for 1 hour with 2 μL IP_1_ conjugated to d2 dye diluted in 13 μL of lysis buffer (Cisbio) and 2 μL terbium cryptate‐labeled anti‐IP_1_ monoclonal antibody also diluted in 13 μL of lysis buffer. 80 μL of each reaction suspension was then transferred and split between 4 wells (20 μL/well) on a 384‐well opaque plate. IP_1_ spectra were collected by exciting samples at 340 nm (bandpass 15 nm). Emission counts were recorded from 600 to 700 nm using a long pass 475 nm filter (FSQ GG475, Newport). Raw IP_1_ signal was calculated as the ratio of fluorescence emissions at 665nm and 620nm. Data were corrected by subtracting the untransfected IP_1_ ratio from cells expressing transfected sensor. Data are presented as a change in IP_1_ ratio following drug treatment. Each experiment included four repeats per condition and was independently repeated at least three times (N > 3).

### 2.6 Statistical analysis

Data are represented as mean values ± SEM. All experiments were repeated for at least three independent trials, with three to six technical repeats per condition (N > 3). Statistical analysis was performed using GraphPad Prism 7.0c (Graphpad Software, Inc). To assess significance across experimental repeats, pooled or un‐pooled data underwent subsequent pairwise ANOVA analysis. Tukey's post hoc test was performed to assess significance when evaluating comparisons between multiple conditions with *P*‐values *P ≤ .05; **P ≤ .01; ***P ≤ .001; ****P ≤ .0001; *****P ≤ .00001.

### 2.7 Nomenclature of targets and ligands

Key protein targets and ligands in this article are hyperlinked to corresponding entries in http://www.guidetopharmacology.org, the common portal for data from the IUPHAR/BPS Guide to PHARMACOLOGY,^20^ and are permanently archived in the Concise Guide to PHARMACOLOGY 2019/20: G protein‐coupled receptors.^21^

## 3 RESULTS

### 3.1 SPASM sensor design

**FIGURE 1:**
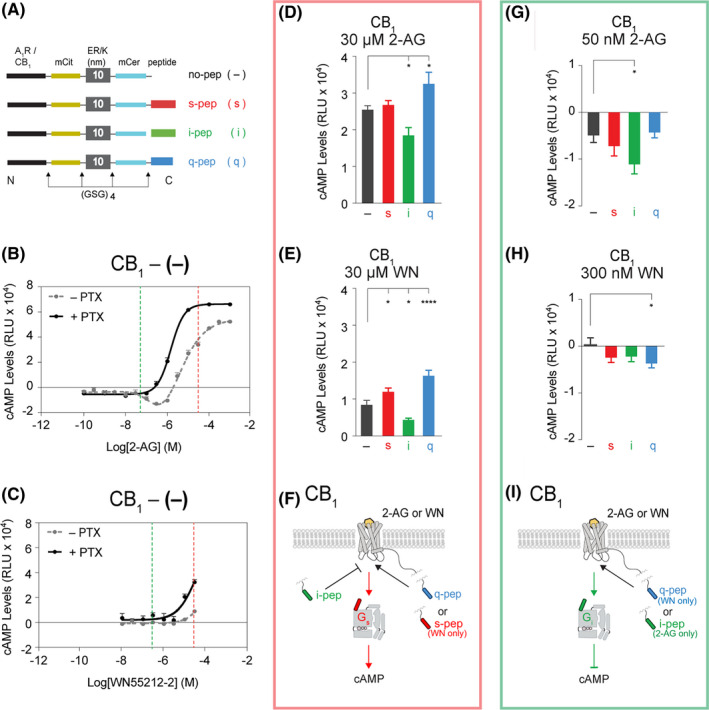
Gα peptides differentially impact Gs and Gi signaling in Cannabinoid (CB_1_) receptors. A, SPASM sensors for characterization of second messenger response. Schematics of the A_1_R and CB_1_ GPCR peptide sensors containing C‐terminal Gα peptides corresponding to s‐, i‐, or q‐ 5α helices separated with Gly‐Ser‐Gly (GSG)_4_ linkers to ensure rotational freedom. The no‐pep (−) construct lacks the Gα C‐terminal peptide. Forskolin‐stimulated cAMP dose‐response curves of B, CB_1_ agonist, 2‐Arachidonoylglycerol (2‐AG), and C, WIN 55,212‐2 mesylate (WN55212‐2) in a CB_1_ no‐pep (−) sensor (representative curves from N = 2 independent biological replicates composed of ≥3 technical repeats each). cAMP levels shown in the absence (*gray line*) and presence (*black line*) of pertussis toxin (PTX) treatment. Ligands potentiate forskolin‐stimulated cAMP accumulation at 30 μmol/L, suggesting Gs bias (B and C, *red dashed lines*). 2‐AG and WIN 55,212‐2 mesylate (WN) inhibit forskolin‐stimulated cAMP at 50 nmol/L and 300 nmol/L, respectively, suggesting Gi bias (B and C, *green dashed lines*). cAMP levels of tethered CB_1_ sensors after stimulation by forskolin and 30 μmol/L 2‐AG (D) or WN (E) (N = 5 independent biological replicates). F, summary of Gα peptide influence on Gs signaling and cAMP production in CB_1_. Inhibition of forskolin‐stimulated cAMP by tethered CB_1_ sensors after stimulation by 50 nmol/L 2‐AG (G) (N = 8 independent biological replicates) or 300 nmol/L WN (H) (N = 6 independent biological replicates). I, summary of Gα peptide influence on Gi signaling and cAMP inhibition in CB_1_. GPCR‐Gα C‐terminal peptide sensors are compared with the no‐pep (−) control. Results are expressed as mean ± SE. ****P < .0001; *P < .05

SPASM sensors were developed for two cognate Gi‐coupled receptors, adenosine A_1_ receptor (A_1_R) and cannabinoid type 1 (CB_1_) (Figure 1A). From N‐ to C‐terminus, each SPASM sensor contains a GPCR, mCitrine (to monitor sensor integrity), 10 nm ER/K linker, mCerulean (for matching receptor expression), and a 27‐amino acid peptide derived from the α5‐helix at the C‐terminus of the Gα subunit (s‐pep, i‐pep, q‐pep, or no‐pep). We chose the 10 nm linker based on previous work, where we found that a shorter linker corresponded to a higher effective concentration of the protein interaction (Figure S1, left).^22^ We had previously shown that a peptide derived from Gαs (s‐pep) could enhance Gs signaling through β2‐AR, and we confirmed this in Figure S1 with β2‐AR producing a significant increase in cAMP when tethered to the s‐pep (Sp) by either a 10 or 20 nm linker.^8^ However, we observed no significant increase in cAMP production by β2‐AR when tethered to s‐pep by a 30 nm linker (Figure S1). We therefore used a 10 nm linker to tether peptides to GPCRs for subsequent experiments, since it appeared that the effective concentrations enforced by either a 10 or 20 nm linker were required to modulate signaling. The Gα C‐terminal peptides have been shown to be essential for activation by the GPCR but do not themselves trigger downstream effectors.^6^, ^8^, ^23^, ^24^, ^25^, ^26^, ^27^ In previous studies we have shown the ability of SPASM sensors to be expressed and localized primarily to the plasma membrane in HEK 293 cells.^28^ Our SPASM sensors are therefore designed to modulate the interaction between the attached receptor (A_1_R or CB_1_) and endogenous G proteins in cells, allowing one to study the impact of the tethered Gα peptides on canonical GPCR signaling.^19^ SPASM A_1_R and CB_1_ constructs lacking a C‐terminal peptide (no‐pep) were used to measure background cAMP and IP_1_ levels and for characterization of ligand dose‐response.

### 3.2 Impact of Gα C‐terminal peptides on cAMP response in the Cannabinoid (CB_1_) Receptor

Cells expressing the CB_1_ sensor display potentiation of forskolin‐stimulated cAMP accumulation with signaling dominated by Gs in response to 30 μmol/L of the CB_1_ agonists 2‐Arachidonoylglycerol (2‐AG) (Figure 1B, *red dashed line*) or WIN 55,212‐2 mesylate (WN) (Figure 1C, *red dashed line*).^10^, ^13^ Representative dose‐response curves with untransfected HEK 293 cells are shown in Figure S2 with stimulation by 2‐AG (Figure S2, *green*) or WN (*purple*). We observed no potentiation of forskolin‐stimulated cAMP accumulation in untransfected HEK 293 cells in response to a range of 2‐AG and WN concentrations (Figure S2), suggesting any potentiation of forskolin‐stimulated cAMP accumulation can be attributed to transfected CB_1_ receptors rather than endogenous receptors in the HEK 293 cells. CB_1_ appeared to inhibit forskolin‐stimulated cAMP accumulation with signaling dominated by Gi in response to 50 nmol/L 2‐AG (Figure 1B, *green dashed line*) or 300 nmol/L WN (Figure 1C, *green dashed line*). To characterize Gi signaling in CB_1_, dose‐response curves were performed for both 2‐AG and WN (Figure 1B and C, respectively) in the presence (*black lines*) or absence (*gray lines*) of pertussis toxin (PTX). cAMP levels increased in response to PTX treatment in 2‐AG‐stimulated CB_1_ (Figure 1B, black line), indicating that cAMP inhibition in the absence of PTX is likely due to signaling through Gi. 2‐AG or WN can be used at high concentrations (30 μmol/L, Figure 1B and C, *red dashed lines*) to characterize the impact of peptides on cAMP stimulation and Gs signaling (Figure 1D and E) and at low concentration (50 or 300 nmol/L, Figure 1B and C, *green dashed lines*) to characterize cAMP inhibition and signaling through Gi in CB_1_ (Figure 1G and H).

We examined the allosteric modulation of Gαs, Gαi, and Gαq peptides on forskolin‐stimulated cAMP accumulation in the promiscuous Gi‐coupled receptor, cannabinoid type 1 (CB_1_). SPASM sensors with s‐, i‐, or q‐pep fusions, in addition to a no‐pep control (−), were expressed in HEK 293 cells as shown previously.^28^ Cells expressing the CB_1_ sensors were treated with high concentrations (30 μmol/L) of 2‐AG (Figure 1D) or WN (Figure 1E) to stimulate cAMP production through the Gs pathway (Figure 1B and C). The q‐pep sensor was found to increase signaling through Gs in CB_1_, as evidenced by a significant increase in cAMP levels (Figure 1D and E, blue bars). This finding in a Gi‐coupled receptor extends our previous results where q‐pep exhibited enhanced signaling in the Gs pathway in Gs‐coupled receptors.^8^ S‐pep sensors also increased signaling through Gs in CB_1_ after stimulation by WN (Figure 1E, red bar). In contrast, the presence of i‐pep inhibited Gs signaling in CB_1_ after stimulation by 2‐AG or WN, decreasing cAMP levels (Figure 1D and E, green bars). These findings are also summarized in the schematic (Figure 1F) with q‐pep (blue) and s‐pep (red) stimulating Gs signaling and i‐pep (green) inhibiting signaling through Gs.

Gα peptides affected signaling through Gi‐mediated inhibition of forskolin‐stimulated cAMP accumulation in CB_1_. To target Gi signaling, HEK 293 cells expressing CB_1_ SPASM sensors were treated with low concentrations of 2‐AG (50 nmol/L) or WN (300 nmol/L), conditions resulting in cAMP inhibition (Figure 1B and C). The i‐pep increased the inhibition of cAMP production after stimulation by 2‐AG (Figure 1G, green bar) compared to the no‐pep (−) sensor. Treatment with WN leads to an increase in Gi signaling with q‐pep but not with i‐pep (Figure 1H, blue bar). The agonist‐dependent enhancement of Gi signaling by both i‐pep and q‐pep is summarized in the schematic (Figure 1I).

### 3.3 Impact of Gα C‐terminal peptides on cAMP inhibition in the Adenosine (A_1_R) Receptor

**FIGURE 2:**
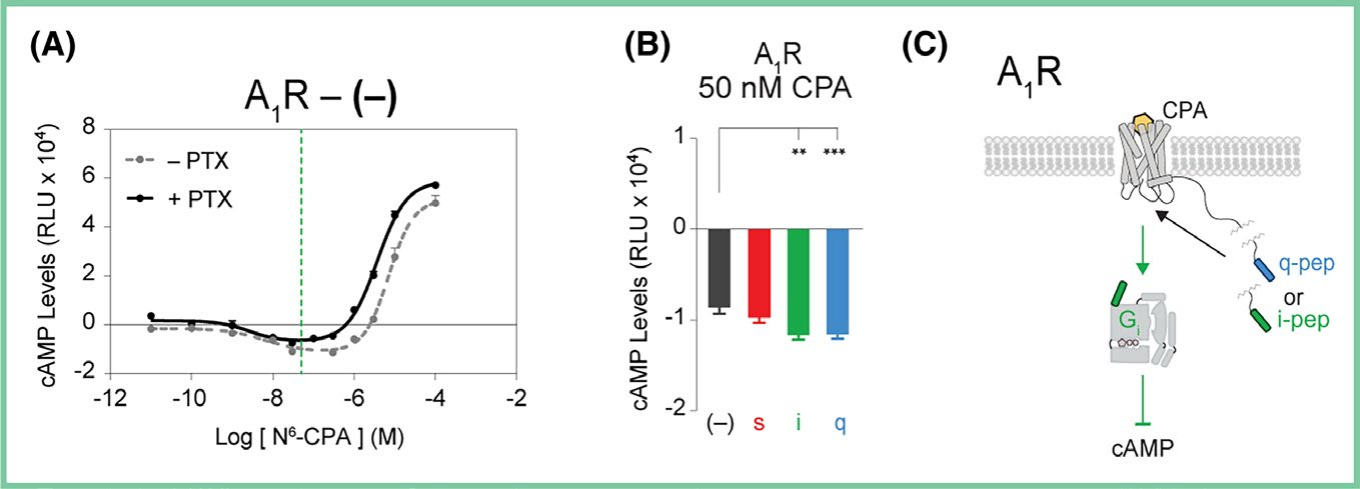
Characterization of cAMP modulation in adenosine receptor (A_1_R) by SPASM sensors. Forskolin‐stimulated cAMP dose‐response curves of (A), A_1_R agonist, N^6^‐Cyclopentyladenosine (CPA). cAMP levels shown in the absence (*gray line*) and presence (*black line*) of pertussis toxin (PTX) treatment. 50 nmol/L CPA inhibits forskolin‐stimulated cAMP, suggesting Gi bias (A, *green dashed line*). B, Inhibition of forskolin‐stimulated cAMP by tethered A_1_R peptide sensors after stimulation by 50 nmol/L CPA. C, Summary of Gα peptide influence on Gi signaling and cAMP inhibition. GPCR‐Gα C‐terminal peptide sensors are compared with the no‐pep (−) control. Results are expressed as mean ± SE. ***P < .001; **P < .01. N = 8 independent biological replicates

Cells expressing the A_1_R no‐pep (−) sensor display Gi‐mediated inhibition of forskolin‐stimulated cAMP accumulation after stimulation by 50 nmol/L of the A_1_R agonist, N^6^‐Cyclopentyladenosine (CPA) (Figure 2A, green dashed line). Pertussis toxin (PTX) treatment inhibits Gi signaling, allowing for differentiation between the Gs‐ and Gi‐mediated effects on cAMP.^29^ cAMP levels increased in response to PTX treatment in CPA‐stimulated A_1_R (Figure 2A, black line), indicating that cAMP inhibition in the absence of PTX is likely due to signaling through Gi. To characterize the impact of different Gα peptides on Gi inhibition of forskolin‐stimulated cAMP accumulation in a promiscuous Gi‐coupled receptor, cells expressing the different A_1_R peptide sensors at equivalent levels were treated with 50 nmol/L of CPA resulting in cAMP inhibition, dominated by Gi (Figure 2B). The i‐pep and q‐pep both increased signaling through Gi in A_1_R after stimulation by CPA, as evidenced by a significant increase in cAMP inhibition (Figure 2B, *green and blue bars*, respectively). To address potential variability in individual sensor response, for each experiment equivalent sensor expression was verified using fluorescence measurements (see methods) and data for all four peptide sensors were collected together (supplemental Figure S3). This phenomenon is summarized in a schematic (Figure 2C) showing the presence of i‐pep (green) and q‐pep (blue) increasing signaling through Gi and inhibiting cAMP.

Despite the potentiation of forskolin‐stimulated cAMP accumulation at high concentrations of CPA (Figure 2A), the cAMP accumulation appears to be the result of stimulation of endogenous HEK 293 cell receptors rather than Gs signaling through A_1_R receptors. Untransfected HEK 293 cells treated with 30 μmol/L CPA showed higher potentiation of forskolin‐stimulated cAMP accumulation than was seen with HEK 293 cells transfected with A_1_R (Figure S4). Under the same conditions, pretreatment with 100 nmol/L of the A_2A_R selective antagonist, SCH 442 416 (SCH), and 1 μmol/L of the A_2B_R selective antagonist, PSB 1115 (PSB), resulted in complete inhibition of cAMP production. The slight decrease in forskolin‐stimulated cAMP accumulation in cells transfected with A_1_R without antagonist pretreatment can likely be attributed to increased Gi signaling by transfected A_1_R receptors. We performed the same control experiments with untransfected HEK 293 cells treated with 30 uM NECA and found equivalent potentiation of forskolin‐stimulated cAMP accumulation as compared to A_1_R‐transfected cells (Figure S4). Pretreatment with A_2A_R and A_2B_R selective antagonists, SCH and PSB, did not change cAMP accumulation in untransfected cells. However, pretreatment with SCH and PSB in A_1_R‐transfected cells reduced cAMP accumulation by 50%. In both cases, treatment with either 30 μmol/L CPA or NECA appears to increase forskolin‐stimulated cAMP accumulation due to endogenous receptors in the HEK 293 cells. A representative dose‐response curve shows potentiation of forskolin‐stimulated cAMP accumulation in untransfected HEK 293 cells in response to a range of CPA and NECA concentrations (Figure S2). We therefore could not characterize the impact of Gα peptides on Gs signaling in A_1_R.

### 3.4 C‐terminal Gα Peptides Inhibit Gq Signaling from Promiscuous Receptors

**FIGURE 3:**
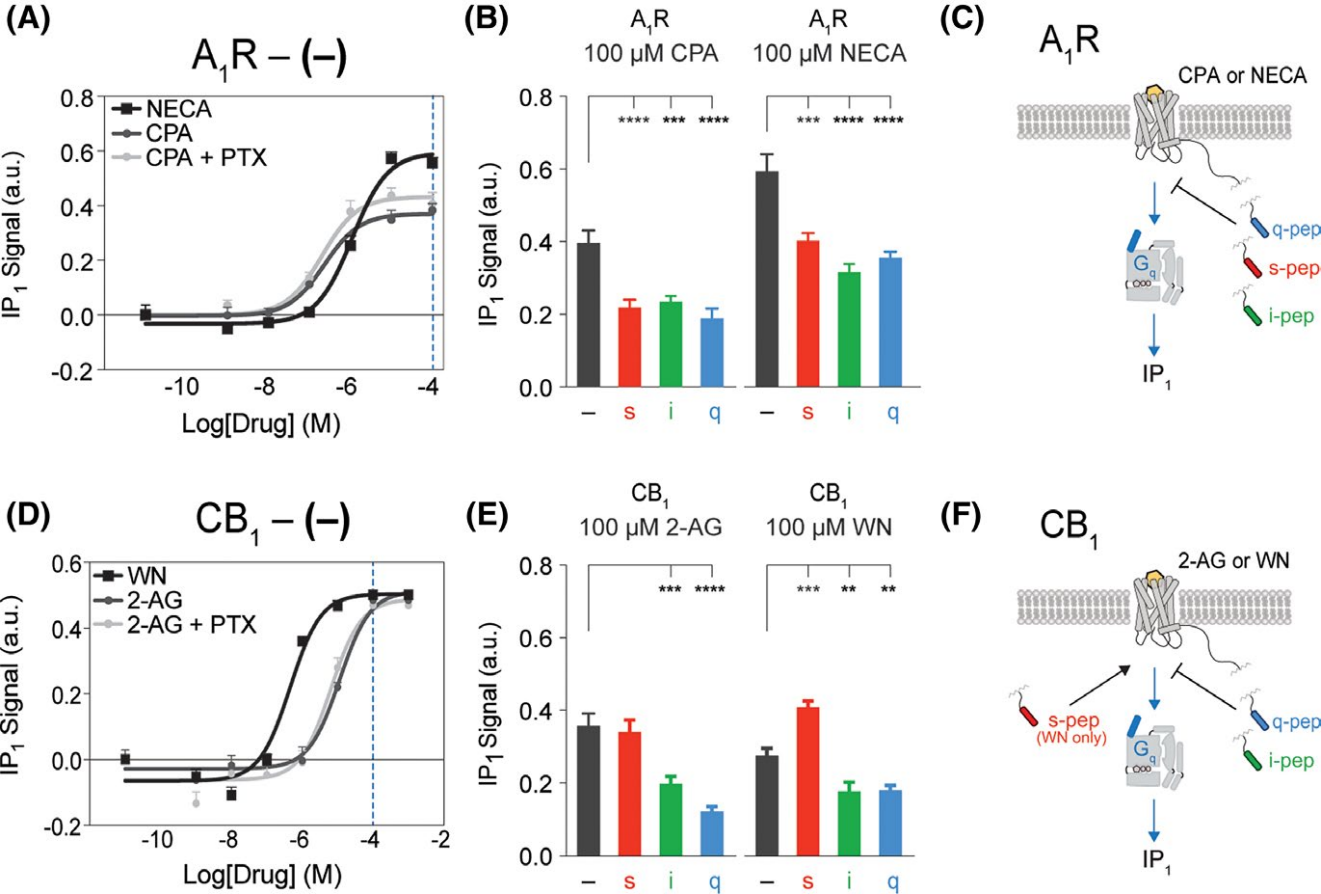
Gαq and Gαi peptides inhibit signaling through Gq. A, IP_1_ dose‐response curve of A_1_R agonists, CPA (*gray lines*) (representative curves from N = 2 independent biological replicates composed of ≥3 technical repeats each) and NECA (*black line*) (N = 3 technical repeats), with A_1_R‐no‐pep (−) sensor. IP_1_ levels shown in the absence (dark *gray line*) and presence (*light gray line*) of pertussis toxin (PTX) treatment. 100 μmol/L CPA or NECA stimulate IP_1_ (A, *blue dashed line*). B, IP_1_ signal from A_1_R after stimulation by 100 μmol/L CPA (left) (N = 3 independent biological replicates) or NECA (right) (N = 4 independent biological replicates) in the presence of different Gα C‐terminal peptides compared to no‐pep (−) control. c, summary of Gα peptide influence on Gq signaling and IP_1_ production in A_1_R. D, IP_1_ dose‐response curve of CB_1_ agonists, 2‐AG (*gray lines*) (N = 5 independent biological replicates) and WN (*black line*) (N = 4 independent biological replicates), with CB_1_‐no‐pep (−) sensor. IP_1_ levels shown in the absence (dark *gray line*) and presence (*light gray line*) of pertussis toxin (PTX) treatment. 100 μmol/L 2‐AG or WN stimulate IP_1_ (D, *blue dashed line*). E, IP_1_ signal from CB_1_ Gα C‐terminal peptide sensors after stimulation by 100 μmol/L 2‐AG (left) or WN (right) compared to no‐pep (−) control (N = 3 technical repeats). F, summary of Gα peptide influence on Gq signaling and IP_1_ production in CB_1_. Results are expressed as mean ± SE. ****P < .0001; ***P < .001; **P < .01

Previous work from our lab suggests that the effect of noncanonical G proteins on IP_1_ signaling are more receptor specific.^8^ We found that Gs enhances IP_1_ production and signaling through Gq in the vasopressin receptor (V_1A_‐R) but not the α1 adrenergic receptor (α1‐AR).^8^ In the current study we examined the impact of Gα peptides on Gq signaling and IP_1_ production in A_1_R and CB_1_ receptors. A dose‐response study of NECA (Figure 3A, *black line*) and CPA (*gray lines*) with A_1_R no‐pep (−) sensors revealed maximum IP_1_ signal at 100 μmol/L ligand (*blue dotted line*). To rule out Gβγ‐dependent PLC‐β activation, we performed IP_1_ dose‐response assays in the absence (Figure 3A, dark *gray line*) and presence (*light gray line*) of pertussis toxin (PTX) treatment. Regardless of CPA concentration, no reduction in IP_1_ production was observed in PTX‐treated cells compared to untreated cells, suggesting the observed IP_1_ production is due to A_1_R signaling through the PTX‐insensitive Gq pathway. Additionally, to rule out Gq signaling through endogenous HEK 293 A_2B_R receptors, IP_1_ levels were assessed in untransfected HEK 293 cells after stimulation by 100 μmol/L CPA or NECA (Figure S5). Regardless of pretreatment with 1 μmol/L of the A_2B_R selective antagonist PSB 1115 (PSB), significant IP_1_ production occured in A_1_R transfected cells but not in untransfected HEK 293 cells, suggesting IP_1_ production resulted from Gq signaling through A_1_R and not endogenous A_2B_R (Figure S5). A_1_R SPASM sensors with tethered s‐, i‐, or q‐pep, in addition to a no‐pep (−) sensor lacking a peptide, were expressed in HEK 293 cells to equivalent levels. IP_1_ assays were performed with each of the A_1_R sensor constructs after stimulation by 100 μmol/L CPA (Figure 3B, left) or NECA (right). Constructs containing the s‐pep, i‐pep, or q‐pep inhibited IP_1_ production regardless of ligand, as summarized in the schematic (Figure 3C).

To examine the impact of the Gα peptides on Gq signaling in a second promiscuous receptor, CB_1_, we first performed assays to identify the optimal concentration of ligand to use for characterization. A dose‐response study of WN (Figure 3D, *black line*) and 2‐AG (*gray lines*) on CB_1_ no‐pep (−) sensors revealed maximum IP_1_ signal at 100 μmol/L ligand (*blue dotted line*). To rule out Gβγ‐dependent PLC‐β activation, we performed IP_1_ dose‐response assays in the absence (Figure 3D, dark *gray line*) and presence (*light gray line*) of pertussis toxin (PTX) treatment. Regardless of 2‐AG concentration, no reduction in IP_1_ production was observed in PTX‐treated cells compared to untreated cells, suggesting the observed IP_1_ production is due to CB_1_ signaling through the PTX‐insensitive Gq pathway. An IP_1_ assay was performed on HEK 293 cells expressing SPASM sensors with s‐, i‐, or q‐pep fusions, in addition to a sensor lacking a peptide no‐pep (‐), after stimulation by 100 μmol/L 2‐AG (Figure 3E, *left*) or WN (*right*). Consistent with A_1_R, the i‐pep and q‐pep inhibited signaling through Gq in CB_1_, as evidenced by reduction of IP_1_ signal (Figure 3E, green and blue bars, respectively). The s‐pep significantly enhanced signaling through Gq after stimulation by 100 μmol/L WN (Figure 3E, right, red bar). The influence of i‐pep, q‐pep, and s‐pep on Gq signaling and subsequent IP_1_ production in CB_1_ is summarized in the schematic (Figure 3F). We have summarized these findings in supplemental model Figure S6, highlighting how Gα C‐terminal peptides differentially influence signaling in each of these promiscuous receptors.

## 4 DISCUSSION

In this study we demonstrate the allosteric modulation of two Gi‐coupled receptors, A_1_R and CB_1_, using peptides derived from the C‐terminus of the Gα subunit (G‐peptides). G‐peptides derived from Gαi and Gαq (i‐pep and q‐pep) enhance agonist‐dependent cAMP inhibition, demonstrating their function as positive allosteric modulators of Gi‐coupled signaling. In contrast, i‐pep and q‐pep suppress agonist‐dependent IP_1_ levels suggesting that they function as negative allosteric modulators of Gq‐coupled signaling. Taken together with our previous studies focused on Gs‐coupled receptors, our findings reinforce the potential of G‐peptides to allosterically modulate signaling from class A GPCRs.^8^, ^9^ While allosteric modulation of GPCR signaling has gained prominence to address the need for receptor specificity, efforts have mainly focused on allosteric sites adjacent to the orthosteric ligand‐binding pocket and lipophilic molecules that target transmembrane helices.^2^ In contrast, here we use as G‐peptides as probe molecules to demonstrate allosteric modulation through the GPCR‐G protein binding interface.

The two Gi‐coupled receptors (CB_1_ and A_1_R) examined in this study have also been reported to signal to varying degrees through other G proteins.^10^, ^11^, ^12^, ^13^, ^16^ While traditionally described as a Gi‐coupled receptor, it has been demonstrated that A_1_R can couple to Gs and Gq in response to CPA or NECA, suggesting A_1_R can adopt agonist‐specific conformations arising from small differences in ligand structure leading to differential G protein activation.^11^ However, previous studies emphasize A_1_R signaling through Gi and contradict signaling through Gs.^30^, ^31^ Our data suggest any apparent Gs signaling by A_1_R, measured by potentiation of forskolin‐induced cAMP production, cannot be distinguished from activation of endogenous A_2A_R or A_2B_R receptors by A_1_R agonists. We saw significantly higher potentiation of forskolin‐induced cAMP production in untransfected HEK 293 cells compared to A_1_R‐transfected HEK 293 cells in response to CPA, suggesting CPA is likely stimulating endogenous Gs‐coupled receptors (Figure S4). Further investigation revealed A_2A_R and A_2B_R specific antagonists could inhibit this potentiation of cAMP in untransfected cells, suggesting any potentiation of forskolin‐induced cAMP production likely resulted from stimulation of endogenous A_2A_R or A_2B_R receptors. Therefore, we could not independently examine A_1_R signaling through the Gs pathway. The A_2B_R receptor is also known for signaling through Gq, however, control experiments confirmed Gq signaling likely occurred through A_1_R and not A_2B_R since no significant IP_1_ production was seen in untransfected HEK 293 cells (Figure S5). In accordance with a previous studies, we confirmed CB_1_ did indeed signal through Gs, as no significant potentiation of forskolin‐induced cAMP production was observed in untransfected HEK 293 cells stimulated by the CB_1_ agonists 2‐AG or WN (Figure S2).^10^, ^13^, ^16^ We therefore used CB_1_ to examine the impact of G‐peptides on Gs signaling, with findings consistent with our previous report for the Gs selective β2‐AR receptor (Figure 1D‐F).^8^

Our data contrast with previous studies that report inhibition of GPCR signaling by native cognate G‐peptides.^32^, ^33^, ^34^ In these studies, minigene vectors were used to overexpress cognate G‐peptides in cells at arbitrarily high concentrations, in order to identify and selectively inhibit cognate G protein engagement with the receptor. Accordingly, we have previously shown that high concentrations of cognate G‐peptides (100 μmol/L s‐pep) can competitively inhibit signaling from Gs‐coupled receptors.^8^, ^9^ In contrast, we find that noncognate G‐peptides can bind weakly to the receptor and serve as positive allosteric modulators.^8^, ^9^ While no significant positive allosteric effects were noted in studies with minigene vectors encoding noncognate G‐peptides, these could be attributed to the variation and/or lack of control in expression since saturating levels would result in inhibition.^32^, ^33^, ^34^ To alleviate the confounding effects of G‐peptide concentration, we used the SPASM constructs to provide equivalent effective concentrations of distinct G‐peptides across different receptor‐ligand‐pathway combinations. Furthermore, the ER/K linker in the SPASM sensors provides an effective concentration of approximately 10 μmol/L,^18^ which is significantly lower than our previously reported threshold for competitive inhibition by cognate G‐peptides. Using this technology, we observe differential effects of G‐peptides on distinct pathways emerging from the same receptor. Specifically, while both i‐pep and q‐pep augment Gi‐mediated cAMP inhibition, they suppress IP_1_ accumulation downstream of Gq activation. Given that sensor expression levels were matched between cAMP and IP_1_ assays and the ER/K linked G‐peptides (i‐pep and q‐pep) are presented at equal effective concentrations, it is unlikely that inhibition of Gq signaling stems from a simple competitive inhibition mechanism. Instead, the differential effects of G‐peptides likely stem from the dynamic conformational landscape of GPCRs.^35^, ^36^

We propose a model wherein transient interactions with G‐peptides alter receptor conformation. The receptor does not form a stable ternary complex with the G‐peptide and therefore at low concentrations (10 μmol/L) does not interfere with the kinetics of the receptor‐cognate G protein interaction.^9^ However, the altered receptor conformation triggered by G‐peptide binding impacts ligand efficacy for cognate G protein activation, resulting in positive or negative allosteric modulation of downstream responses. The inability of the G‐peptides, especially those derived from noncognate G proteins, to form stable interactions with the receptor has been previously observed in A_1_R‐Gi fusions.^37^ The lack of stable ternary complex formation with noncognate G proteins has been suggested as a kinetic proofreading mechanism to prevent noncognate GPCR‐G protein coupling.^37^ Nonetheless, we have previously shown that both cognate and noncognate G‐peptide interactions influence receptor conformation.^9^ Transient interactions of the G‐peptide at the cognate G protein binding site on the receptor stabilize a distinct receptor conformational state. This conformational state persists following G‐peptide dissociation enabling increased efficacy of subsequent cognate G protein coupling and enhanced downstream signaling.^9^ Given that the G‐peptide and cognate G protein share the same binding site, albeit staggered in time, we propose that the G‐peptides function as allokairic modulators (AKMs) of cognate GPCR signaling. Allokairy is an established concept in enzymatic reactions, wherein increased substrate concentrations can increase maximal reaction rates, especially if the substrate stabilizes a distinct active enzyme conformation.^38^ AKMs can bind asynchronously with the orthosteric ligand and rely on temporally persistent conformational states of the enzyme to exert their effects.^9^ G‐peptides as AKMs provide access to the entire GPCR‐G protein interaction interface for allosteric modulation, without necessarily competing with cognate G protein coupling. Targeting the GPCR‐G protein interface offers the potential to enhance receptor specificity, especially given the three intrinsically disordered loop regions with considerable isoform specific sequence homogeneity.

## 5 ETHICS STATEMENT

No animals, human tissue, human volunteers, or patients were used in this study.

## AUTHORSHIP CONTRIBUTIONS

Touma, Malik, and Sivaramakrishnan participated in research design. Touma, Malik, and Gupte conducted experiments.

Touma, Malik, Gupte, and Sivaramakrishnan performed data analysis.

Touma and Sivaramakrishnan wrote manuscript.

## Supporting information

Figure S1‐S6

## Data Availability

Additional information and requests for data and/or reagents should be directed to the corresponding author, Dr Sivaraj Sivaramakrishnan. Please contact sivaraj@umn.edu.
