# Supplementary material for: Allosteric modulation of adenosine A1 and cannabinoid 1 receptor signaling by G‐peptides
Source: Pharmacol Res Perspect. 2020 Oct 30;8(6):e00673. doi: 10.1002/prp2.673 (PMC7596666; doi:10.1002/prp2.673)
Supplement: Supplementary file 1 — Figure S1‐S6 [file PRP2-8-e00673-s001.docx]

**SUPPLEMENTAL**

**FIGURE S1**


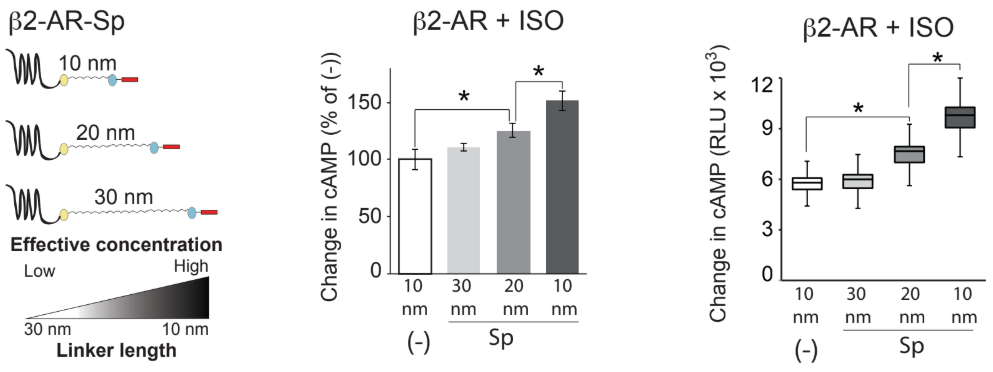


**FIGURE S1.** **Length of tether and modulation of signaling in β2-AR.** *Left*, schematic of Gαs C-terminal peptide (*red*) fused to β2-AR via 10, 20, or 30 nm ER/K linkers. Schematic shows effective concentration of protein interaction increasing as linker length decreases. *Right*, relative increase in cAMP production after isoproterenol (ISO) stimulation of 30, 20, and 10 nm β2-AR-s-pep (Sp) sensors compared to a β2-AR sensor lacking peptide (-). Results are represented as percent change from β2-AR no-pep (-) and expressed as mean ±S.E.M. *, p<0.05. N=9 technical repeats from 3 independent biological replicates.

**
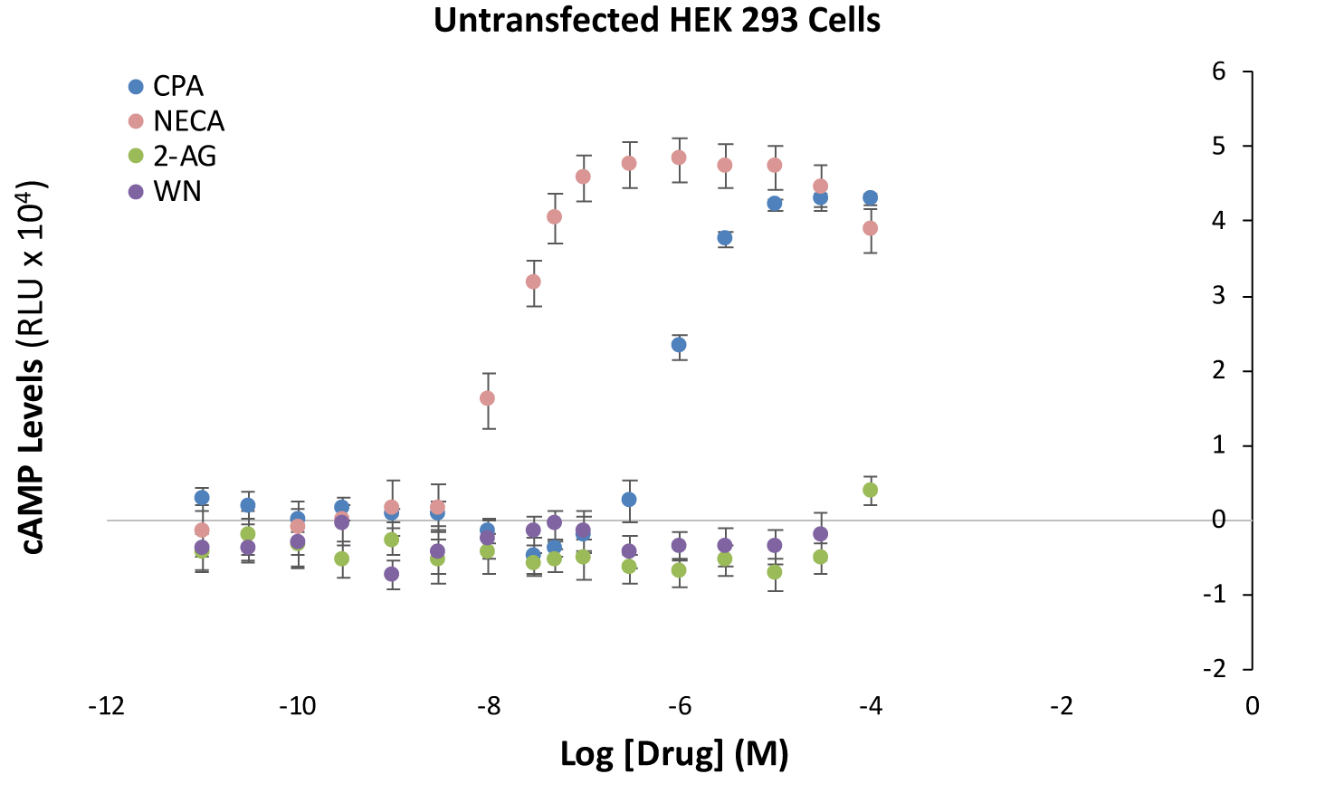
FIGURE S2**

**FIGURE S2.** **cAMP stimulation by endogenous HEK 293 cell receptors.** Representative forskolin-stimulated cAMP dose response curves of untransfected HEK 293 cells shown after stimulation by A1R agonists, CPA (*blue*) and NECA (*pink*), or CB_1_ agonists, 2-AG (*green*) and WN (*purple*). Results expressed as ±S.E.M. from 3 technical replicates.

**FIGURE S3**


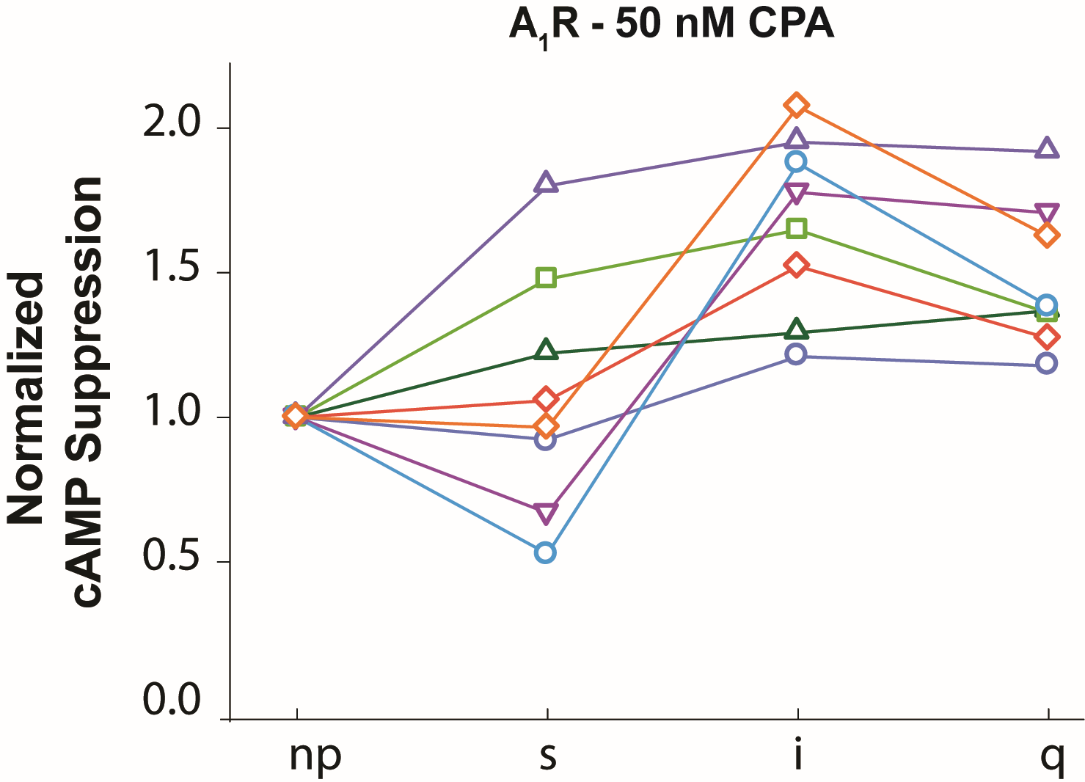


**FIGURE S3. Characterization of cAMP modulation in adenosine receptor (A_1_R) by SPASM sensors.** Inhibition of forskolin-stimulated cAMP by tethered A_1_R peptide sensors, s-, i-, and q-pep, normalized to no-pep (np) control, after stimulation by 50 nM CPA. Each independent experiment (total of n=8 experiments) is represented by a separate color. For each experiment, data for all four peptide sensors were collected together.

**
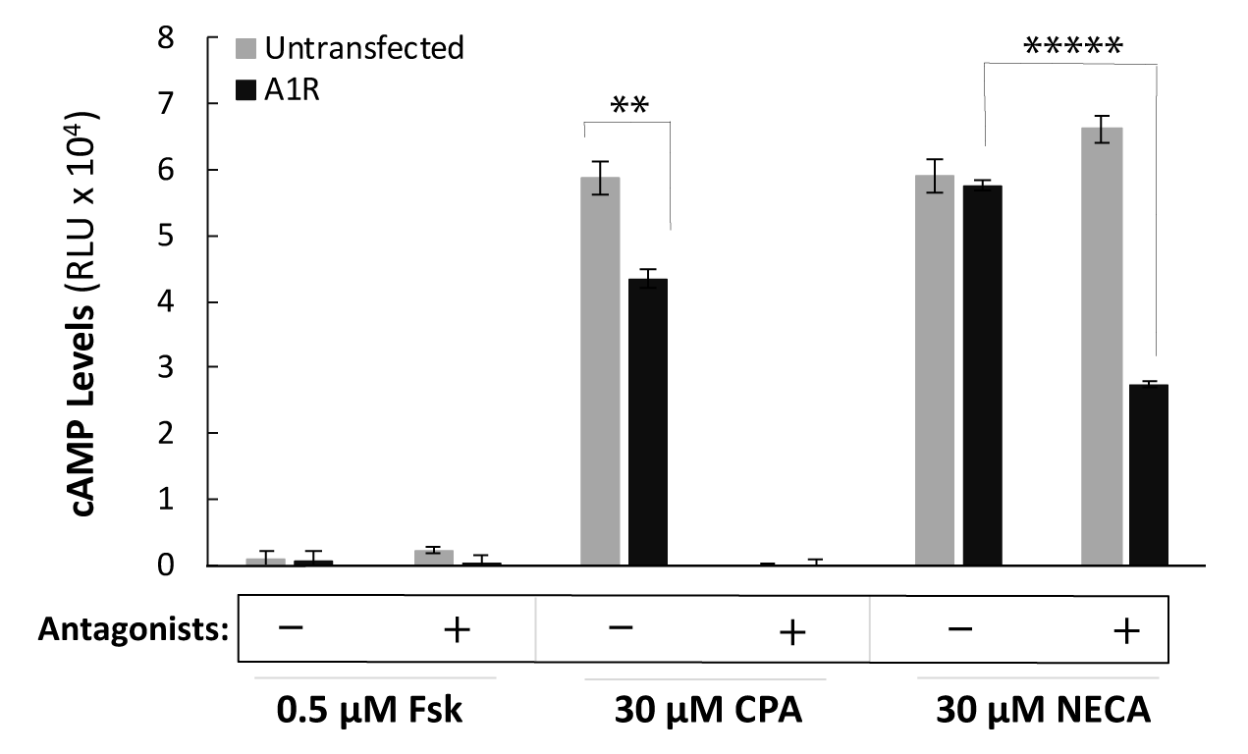
FIGURE S4**

**FIGURE S4.** **cAMP stimulation by endogenous HEK 293 cell receptors and adenosine receptor (A_1_R).** cAMP levels of untransfected HEK 293 cells (gray bars) and HEK 293 cells transfected with A1R (black bars). cAMP levels shown after stimulation by 0.5 μM forskolin (fsk) alone (*left*), 0.5 μM fsk + 30 μM CPA (*middle*), and 0.5 μM fsk + 30 μM NECA (*right*). Conditions with (+) antagonists were pre-treated with 100 nM of the A_2A_R selective antagonist, SCH 442416 (SCH), and 1 μM of the A_2B_R selective antagonist, PSB 1115 (PSB). Results are expressed as mean ±S.E.M. *****, p<0.00001; **, p<0.01. N=3 independent biological replicates.

**FIGURE S5**

**
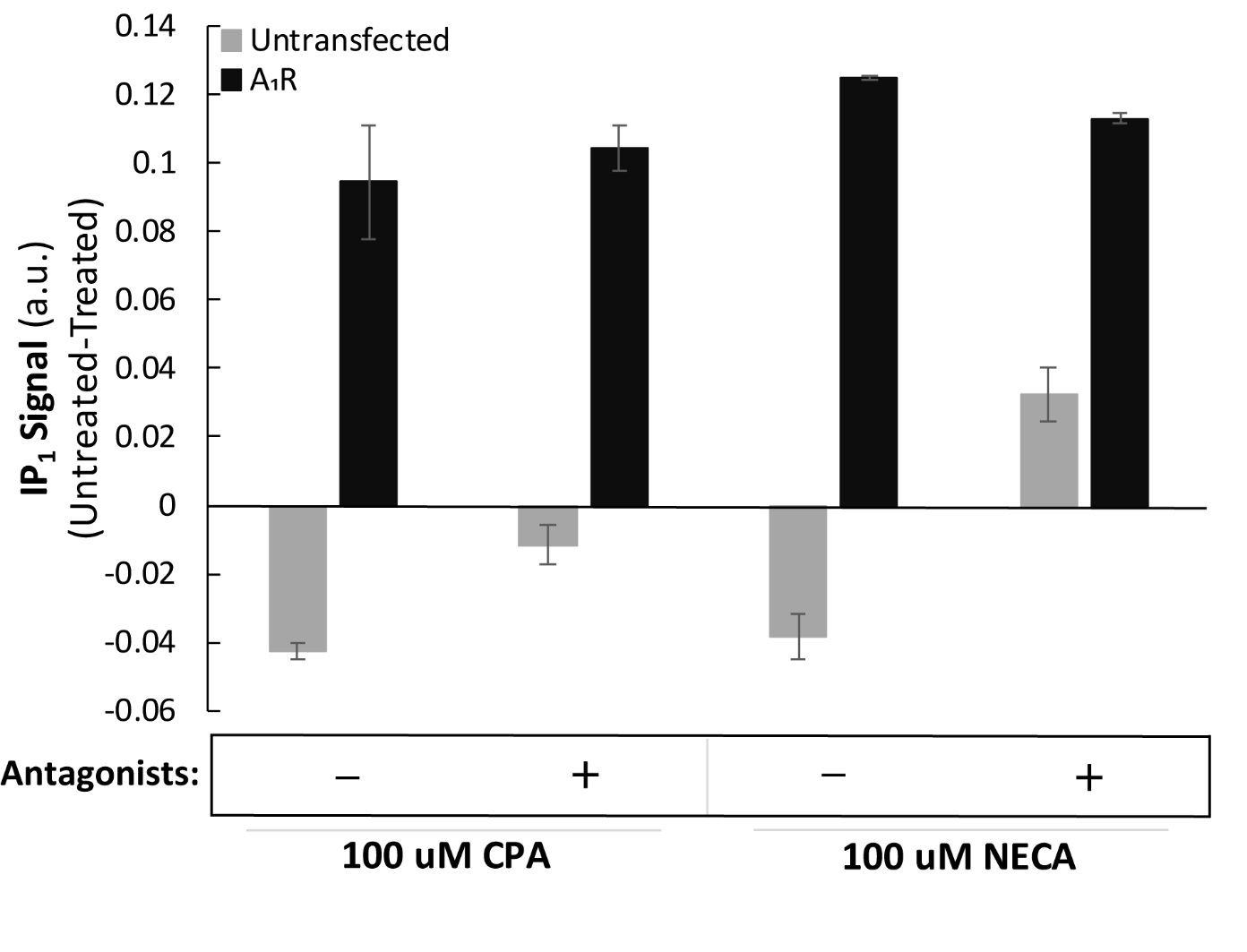
FIGURE S5.** **IP_1_ production by endogenous HEK 293 cell receptors and adenosine receptor (A_1_R).** IP_1_ levels of untransfected HEK 293 cells (gray bars) and HEK 293 cells transfected with A_1_R (black bars). IP_1_ levels shown after stimulation by 100 μM CPA (*left*), and 100 μM NECA (*right*). Conditions with (+) antagonist were pre-treated with 1 μM of the A_2B_R selective antagonist, PSB 1115 (PSB). Results are expressed as mean ±S.E.M. N=9 independent biological replicates.

**FIGURE S6**


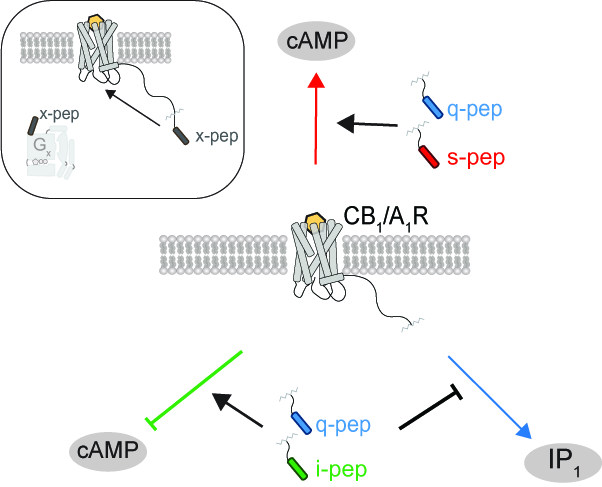


**FIGURE S6.** **Model of Gα peptide influence on GPCR-G protein signaling.** *Inset*, SPASM sensor with attached peptide (x-pep) shown next to G-protein containing C-terminal Gα peptide (x-pep). *Above*, model of Gα peptides (s-pep, i-pep, or q-pep) that modulate Gs (*red arrow*), Gi (*green inhibitory arrow*), or Gq (*blue arrow*) signaling pathways in CB_1_ or A_1_R receptors after stimulation by different agonists. Q-pep and s-pep enhance Gs signaling. Q-pep and i-pep enhance Gi signaling while inhibiting Gq signaling.
